# Rab31-dependent regulation of transforming growth factor ß expression in breast cancer cells

**DOI:** 10.1186/s10020-021-00419-8

**Published:** 2021-12-14

**Authors:** Susanne Soelch, Nathalie Beaufort, Daniela Loessner, Matthias Kotzsch, Ute Reuning, Thomas Luther, Thomas Kirchner, Viktor Magdolen

**Affiliations:** 1 Clinical Research Unit, Department of Obstetrics and Gynecology, Technische Universität München, Ismaninger Str. 22, 81576 Munich, Germany; 2 Institute for Stroke and Dementia Research, Klinikum Der Universität München, Munich, Germany; 3 Leibniz-Institut für Polymerforschung Dresden e.V, Dresden, Germany; 4 Faculty of Engineering and Faculty of Medicine, Nursing and Health Sciences, Monash University, Melbourne, VIC Australia; 5 Medizinisches Labor Ostsachsen, Dresden, Germany

**Keywords:** TGF-ß signaling, GTPase, Rab31, Breast cancer, EMT

## Abstract

**Background:**

The small GTP-binding protein Rab31 plays an important role in the modulation of tumor biological-relevant processes, including cell proliferation, adhesion, and invasion. As an underlying mechanism, Rab31 is presumed to act as a molecular switch between a more proliferative and an invasive phenotype. This prompted us to analyze whether Rab31 overexpression in breast cancer cells affects expression of genes involved in epithelial-to-mesenchymal transition (EMT)-like processes when compared to Rab31 low-expressing cells.

**Methods:**

Commercially available profiler PCR arrays were applied to search for differentially expressed genes in Rab31 high- and low-expressing CAMA-1 breast cancer cells. Differential expression of selected candidate genes in response to Rab31 overexpression in CAMA-1 cells was validated by independent qPCR and protein assays.

**Results:**

Gene expression profiling of key genes involved in EMT, or its reciprocal process MET, identified 9 genes being significantly up- or down-regulated in Rab31 overexpressing CAMA-1 cells, with the strongest effects seen for *TGFB1*, encoding TGF-ß1 (> 25-fold down-regulation in Rab31 overexpressing cells). Subsequent validation analyses by qPCR revealed a strong down-regulation of *TGFB1* mRNA levels in response to increased Rab31 expression not only in CAMA-1 cells, but also in another breast cancer cell line, MDA-MB-231. Using ELISA and Western blot analysis, a considerable reduction of both intracellular and secreted TGF-ß1 antigen levels was determined in Rab31 overexpressing cells compared to vector control cells. Furthermore, reduced TGF-ß activity was observed upon Rab31 overexpression in CAMA-1 cells using a sensitive TGF-ß bioassay. Finally, the relationship between Rab31 expression and the TGF-ß axis was analyzed by another profiler PCR array focusing on genes involved in TGF-ß signaling. We found 12 out of 84 mRNAs significantly reduced and 7 mRNAs significantly increased upon Rab31 overexpression.

**Conclusions:**

Our results demonstrate that Rab31 is a potent modulator of the expression of TGF-ß and other components of the TGF-ß signaling pathway in breast cancer cells.

## Background

Rab-GTPases of the large Ras superfamily of small GTP-binding proteins are key regulators of membrane vesicle trafficking, membrane targeting and fusion in eukaryotic cells (Goud and Gleeson 2010; Stenmark 2009). Up to now, more than 60 different human Rab proteins have been identified, and a growing number of Rab and Rab-related proteins has been functionally characterized (Homma et al. 2020; Pylypenko et al. 2018; Singan et al. 2012). The importance of Rab pathways for intracellular transport processes becomes apparent by their involvement in a number of diseases, such as immunodeficiencies, neurological disorders, and cancer progression, attributed to dysregulation of Rab protein function (Cheng et al. 2005; Chia and Tang 2009; Mitra et al. 2011; Pan et al. 2016; Tzeng and Wang 2016).

Rab31 (also known as rab22B) belongs to the Rab-II supergroup, which encompasses 9 human members. It is ubiquitously expressed in normal human tissue (Bao et al. 2002; Kloepper et al. 2012) and is mainly localized in the trans-Golgi network and endosomes (Bao et al. 2002; Ng et al. 2007; Rodriguez-Gabin et al. 2001). In these cellular compartments, it regulates vesicle transport from the Golgi apparatus to early and late endosomes, e.g. the transport of mannose-phosphate receptors (Rodriguez-Gabin et al. 2009), or in retrograde transport from the plasma membrane into late endosomes, e.g. in case of the EGF receptor (EGFR) (Chua and Tang 2015). Rab31 silencing inhibits trafficking of EGR-bound EGFR to the late endosomes, and, thus, may prolong EGFR signaling. Contrariwise, Rab31 overexpression enhances EGFR trafficking to late endosomes resulting in an enhanced degradation of the receptor (Chua and Tang 2015).

Dysregulated expression of Rab31 has been observed in benign conditions, such as skin diseases, but is also involved in multiple aspects of tumor progression in various types of cancer, including breast, ovarian, cervical and liver cancer as well as glioblastoma (for a review see [Kotzsch et al. 2016]). In breast cancer tissue, Rab31 was reported to be strongly up-regulated (Kotzsch et al. 2008). Elevated Rab31 mRNA transcript levels were significantly associated with shorter distant metastasis-free survival and overall survival in a cohort of 280 untreated, lymph node-negative breast cancer patients. Since Rab31 mRNA independently contributed to the base multivariate models, it may serve as a prognostic marker of disease recurrence (Kotzsch 2008). In addition, the C-terminal subunit of mucin-1 (MUC1-C), in concert with estrogen receptor α (ERα), was identified to activate Rab31 gene expression in breast cancer, whereby Rab31 upregulation, in turn, results in elevated MUC1-C levels, most likely by reducing its lysosomal degradation. In line with these observations, Rab31 and MUC1-C are co-expressed in ER + tumor tissues (Jin et al. 2012). Subsequently, protein expression of Rab31 and mucin-1 was analyzed in tumor tissue extracts of ER + breast cancer and was found to be correlated with patients’ prognosis (Kotzsch et al. 2017). These results strongly suggest that, in ER + breast cancer patients, high Rab31 antigen levels in tumor tissue are associated with a high proliferative status, and Rab31 is an independent biomarker for poor prognosis (Kotzsch et al. 2017).

Using breast cancer cell transfectants displaying different Rab31 mRNA expression levels, the modulatory role of Rab31 in tumor biologically-relevant processes was investigated*.* Increased Rab31 protein levels were associated with enhanced proliferation of breast cancer cells, reduced cell adhesion and decreased invasive capacity in vitro and in vivo (Grismayer et al. 2012). These results suggest that Rab31, depending on its expression levels, is involved in controlling the interchange between a proliferative and an invasive phenotype.

This proposed function of Rab31 to act as a molecular switch between proliferation and invasion as well as its ability to affect the activity of receptors of important signaling pathways, such as ER or EGFR, raised the question whether Rab31 may modulate the expression of other tumor-associated factors in these processes. Cell proliferation, adhesion, motility and invasion are strongly associated with the developmental program of the epithelial-to-mesenchymal transition (EMT). Originally identified as a program during embryonic development in which cells undergo dynamic changes from epithelial to mesenchymal phenotypes, it became clear that EMT-like processes are driving cancer progression as well. Many different variations of cancer-associated EMT-like programs exist and, thus, cannot be strictly and accurately defined by specific sets of markers (Yang et al. 2020). It is reasonable to assume that some of the genes previously linked to EMT-like processes may also play a role in the effects mediated by Rab31 in breast cancer cells.

In the present study, we used an EMT profiler PCR array which quantifies expression of key genes involved in EMT or its reciprocal process mesenchymal-to-epithelial transition (MET), to analyze whether Rab31 overexpression in breast cancer cells affects expression of some of those genes compared to Rab31 low-expressing cells. We identified *TGFB1* as the most strongly regulated gene in ER + CAMA-1 breast cancer cells (> 25-fold down-regulation in Rab31 overexpressing cells). The relationship between Rab31 expression and the TGF-ß signaling axis was further underscored by the identification of several other genes encoding members of the TGF-ß signaling pathway, which are also controlled by the Rab31 expression level in CAMA-1 cells.

## Methods

### Culture of adherent cells

The human breast adenocarcinoma cell lines CAMA-1 and MDA-MB-231 (American Type Culture Collection, Manassas, VA) were grown in Dulbecco’s modified Eagle medium (DMEM; Gibco BRL, Eggstein, Germany) supplemented with 10% fetal calf serum (FCS; Gibco BRL) and 10 mM HEPES (Gibco BRL; complete medium). Cell transfection with the Rab31 expression vector pRcRSV-Rab31 and the vector control pRcRSV, respectively, and selection of transfectants was performed as previously described (Grismayer et al. 2012).

### 3D cell culture

QGel (QGel, Ecublens VD, Switzerland), a synthetic polyethylene glycol (PEG)-based hydrogel matrix, mimics key features of the natural extracellular microenvironment of cells in vivo and provides a microenvironment for in vitro 3D cell culture. Hydrogels were prepared according to the manufacturer’s instructions (QGel). Briefly, one vial of QGel powder was reconstituted with 400 µl of buffer A. Then, 1 × 10^5^ cells were added to the reconstituted hydrogel in 100 µl phenol red-free complete medium and mixed for 10 s. Droplets (30 µl) of the precursor solution were pipetted on Sigmacote (Sigma-Aldrich, Taufkirchen, Germany; [Ehrbar et al. 2007]) coated glass slides with two 1.5 mm spacers and covered with a second glass slide. For polymerization, droplets were incubated for 45 min at 37 °C. Subsequently, phosphate-buffered saline (PBS, pH 7.4) was applied between the slides to moisten and lift the hydrogel discs for transfer into 48-well plates with complete medium. After 4 h at 37 °C the medium was changed. During 3D cell culture (14 days), the medium was changed every three days. Proliferation analysis of Rab31-transfected and vector cells was performed using both AlamarBlue and CyQuant-based assays as previously described (Loessner et al. 2016).

### Preparation of cell lysates and supernatants

Adherent CAMA-1 and MDA-MB-231 cells were detached and washed with PBS. Cell pellets were disrupted by two freezing and thawing cycles, followed by solubilization of Rab31 antigen in lysis buffer (20  mM Tris–HCl, 125 mM NaCl, pH 7.6, containing 1% [v/v] Triton X-100, and the “Complete” protease inhibitor cocktail, Sigma-Aldrich). After incubation for 60 min on ice, lysates were centrifuged at 2000×*g* for 10 min at 4 °C, the supernatant was collected and used directly for further experiments or frozen at −20 °C. Prior to TGF-ß Western blot analyses, ELISA and activity assays, cell culture supernatants were concentrated 16-fold in a spin column (10,000 MW cut off, Vivaspin 6; Sartorius Stedim, Göttingen, Germany) via centrifugation at 4500×*g* at 4 °C. The total protein content in lysates and supernatants was determined using the BCA Protein Assay Kit according to the manufacturer’s instructions (Thermo Scientific, Dreieich, Germany).

### Western blot analysis

Proteins were separated by electrophoresis on 12% (w/v) polyacrylamide gels (SDS-PAGE), and transferred to polyvinylidene fluoride membranes (Immobilon; Millipore Corporation, Bedford, MA) in either a semi-dry or a wet blotting device (Biometra, Göttingen, Germany; Bio-Rad, Hercules, CA). After blotting, membranes were washed once in Tris-buffered saline (TBS), pH 7.4, containing 0.1% (v/v) Tween-20 (TBS-T), and incubated for blocking in TBS-T, containing 5% (w/v) dried skimmed milk for 60 min at room temperature (RT), followed by overnight incubation with a polyclonal Rab31-directed rabbit antibody (RT3-IgG; [Grismayer et al. 2012]) or with a polyclonal goat antibody directed against the so called latent-associated protein (LAP) domain of human TGF-ß1 (R&D Systems Wiesbaden, Germany), diluted in blocking buffer. After washing with TBS-T, the secondary peroxidase-conjugated goat anti-rabbit or rabbit anti-goat IgG (Jackson ImmunoResearch Lab, West Grove, PA) diluted in TBS-T, containing 1% (w/v) milk powder was applied for 60 min at RT followed by three washes with TBS-T. Proteins were visualized by a chemiluminescent reaction using ECL reagents or Immobilon Western Chemiluminescent HRP Substrate according to the manufacturers' recommendations (Thermo Scientific).

### Rab31 ELISA

The Rab31 antigen concentration in cell lysates was determined using a sandwich ELISA format as described previously (Grismayer et al. 2012; Kotzsch et al. 2017). Briefly, 96-well plates (MaxiSorp™; Nunc, Wiesbaden, Germany) were coated overnight with monoclonal antibody M01 (Novus Biologicals, Wiesbaden, Germany) diluted in coating buffer (15 mM Na_2_CO_3_, 33 mM NaHCO_3_, pH 9.6). After washing twice with washing buffer (PBS, containing 0.5% [v/v] Tween 20, pH 7.6), plates were blocked with blocking solution (washing buffer containing 2% [v/v] neonatal calf serum; Gibco BRL) for 30 min at 37 °C. Thereafter, plates were incubated with cell lysates diluted in sample buffer (50 mM Tris/HCl, 100 mM NaCl, 0.2% [v/v] Triton X-100, 1% [w/v] bovine serum albumin [BSA], pH 7.6) for 90 min at 37 °C. Two-fold serial dilutions of Rab31-His protein (Grismayer et al. 2012) in sample buffer covering a concentration range of 0.15 to 5 ng/ml were used as standard antigen. After washing, plates were incubated with polyclonal antibody RT3-IgG (Grismayer et al. 2012) for 90 min at 37 °C followed by incubation with secondary peroxidase-labeled goat anti-rabbit IgG (Novus Biologicals) for 60 min at 37 °C. Finally, plates were washed and the peroxidase reaction was initiated by addition of 3,3’,5,5’-tetramethylbenzidine/H_2_O_2_ (TMB; K & P Laboratories, Gaithersburg, MD). After 20 min at RT, the reaction was stopped by addition of 0.5 M H_2_SO_4_, and the optical density was measured at 450 nm using a multichannel microplate reader (SLT Spectra, Salzburg, Austria). Absorbance values were converted into ng/ml of Rab31 using the standard curve as reference. The Rab31 concentration is expressed as ng Rab31 per mg of total protein content (ng/mg) of cell lysates.

### Immunocytochemistry

For immunofluorescence analysis, 2 × 10^4^ cells per well were seeded in human fibronectin-coated (5 µg/ml; BD-Bioscience, Bedford, MA) 8-well chamber glass slides (Permanox-type Lab-Tek slides; Nunc, Roskilde, Denmark) and grown overnight. Cell monolayers were fixed with 4% paraformaldehyde in PBS, pH 7.4, for 15 min at RT, washed once with PBS and permeabilized using 0.025% (w/v) saponin in PBS. For blocking, cells were incubated with PBS, containing 2% (w/v) BSA for 30 min at RT. Thereafter, cells were probed with polyclonal rabbit anti-Rab31 antibody C15 (Santa Cruz, Heidelberg, Germany) diluted in PBS containing 1% (w/v) BSA for 90 min at RT. After washing, cells were incubated with the secondary, fluorochrome-coupled polyclonal antibody (goat anti-rabbit-Alexa Fluor 488 IgG; Life Technologies, Darmstadt, Germany), diluted in PBS containing 1% BSA for 45 min at RT in the dark. After final washings with PBS, cells were mounted in PBS and fluorescence intensity was evaluated by confocal laser scanning microscopy (CLSM; Zeiss Axio Observer Z1, Zeiss, Oberkochen, Germany). In order to convert fluorescence staining intensity into colors, the look-up table “glowOv/Un LUT” provided with the CLSM scanning software (Zeiss, black edition, version 7.0). Negative control staining was done with the secondary Alexa Fluor 488-conjugated IgG only.

### TGF-β1 ELISA

TGF-ß1 concentration in cell lysates and cell culture supernatants was determined using an ELISA kit (R&D Systems) according to the manufacturer’s instructions. This ELISA has been broadly used to measure TGF-ß1 protein levels not only in cell supernatants but also in cell extracts (Schmidt et al. 2002; Barsotti et al. 2013; Manokawinchoke et al. 2019). To activate latent TGF-ß1 to its immunoreactive form, cell lysates or culture supernatants were acidified with 1 M HCl prepared in 0.5 M HEPES for 10 min at RT, followed by neutralization with 1.2 M NaOH in 0.5 M HEPES.

### TGF-ß activity assay

To measure active TGF-ß, embryonic fibroblasts derived from TGF-ß-deficient mice, and stably transfected with an expression plasmid encoding for the secreted alkaline phosphatase (SEAP) under the control of a Smad2-responsive promoter were used (MFB-F11 cells; [Beaufort et al. 2014; Broekelmann et al 2020; Johnston et al. 2017; Tesseur et al. 2006). A density of 2 × 10^4^ MFB-F11 cells per well were seeded in 96-well plates (Corning Inc., Corning, NY) and grown for 24 h at 37 °C. After washing with PBS, cells were serum-starved in FCS-free DMEM for 2 h at 37 °C. Concentrated supernatants of CAMA-1 cells (see above) or concentrated fresh culture medium (negative control) were either left untreated or were heated for 10 min at 80 °C to activate TGF-β. Supernatants were added to MFB-F11 cells in two volumes of FCS-free DMEM and incubated for 20 h at 37 °C. Samples were collected and SEAP was measured using a chemiluminescence detection kit (Great EscAPe SEAP; Clontech, Mountain View, CA) according to the manufacturer’s instructions.

### RNA isolation

Total RNA of transfected CAMA-1 and MDA-MB-231 breast cancer cells was prepared using the RNeasy Mini kit (Qiagen, Hilden, Germany) in combination with the QIAcube (Qiagen) fully automated spin column system according to the manufacturer’s instructions. RNA concentration and purity were determined using the Nano Drop ND1000 (Peqlab, Erlangen, Germany) spectrophotometer with the Nano Drop software (Fisher Scientific, Mannheim, Germany). RNase-free water (Qiagen) was used as blank solution.

### RT^2^ profiler PCR arrays

RNA (input of total RNA: 1.0 µg per reaction) was reverse transcribed using the RT^2^ First-Strand kit (Qiagen). Subsequently, profiler array-based quantitative PCR analysis was performed, following the instructions of the supplier, using the RT^2^ SYBR Green PCR Mastermix (Qiagen) and primer pre-coated RT^2^ Profiler PCR array plates (Qiagen) encompassing 84 pathway-focused genes per plate. Two different human-specific profiler PCR arrays were screened: pathways related to EMT signaling and the TGF-β/bone morphogenic protein (BMP), each on two independent biological replicates.

### Quantitative PCR

For quantification of mRNA expression levels of individual genes, total RNA was isolated from at least 3 independent biological replicates and reverse transcribed using the cloned AMV first-strand cDNA Synthesis Kit (Invitrogen, Darmstadt, Germany) and random hexamer primers according to the manufacturer’s instructions. The generated cDNA was diluted 1:5 with RNase-free water to an estimated final cDNA concentration of 10 ng/µl. The Brilliant III QPCR Master Mix with low ROX (Agilent Technologies, Waldbronn, Germany) in combination with FAM-labeled probes and primers (Life Technologies) and the Stratagene Mx3005P qPCR System (Agilent Technologies) were used for qPCR quantification of *RAB31*, *TGFB1* and *HPRT1*. The expression levels of *RAB31* and *TGFB1*, respectively, were normalized to the housekeeping gene hypoxanthine guanine phosphoribosyl transferase 1 (*HPRT*). The concentration of cDNA per qPCR run was 30 ng. For validation of the expression levels of the candidate genes identified in the TGF-ß1/BMP pathway profiler array screen, qPCR was performed using Universal Probe-Libray Probes (Roche, Mannheim, Germany), Brilliant III Ultra-Fast SYBR Green Low ROX Mastermix (Agilent Technologies) and 25 ng cDNA as template. Here, expression levels of the target genes were normalized to 18S RNA expression, using 18S-specific primers and a Universal Probe Library probe.

### Statistical analyses

Data were analyzed for statistical significance using the Mann–Whitney U-test using the StatView 5.0 statistical packet (SAS Institute, Cary, NC). Differences with *P*-values ≤ 0.05 were considered as statistically significant (*). *P*-values ≤ 0.01 and ≤ 0.001 were marked (**) and (***), respectively.

## Results

### Rab31 overexpression in CAMA-1 breast cancer cells leads to increased proliferation of microtumor-like spheroids

**Fig. 1:**
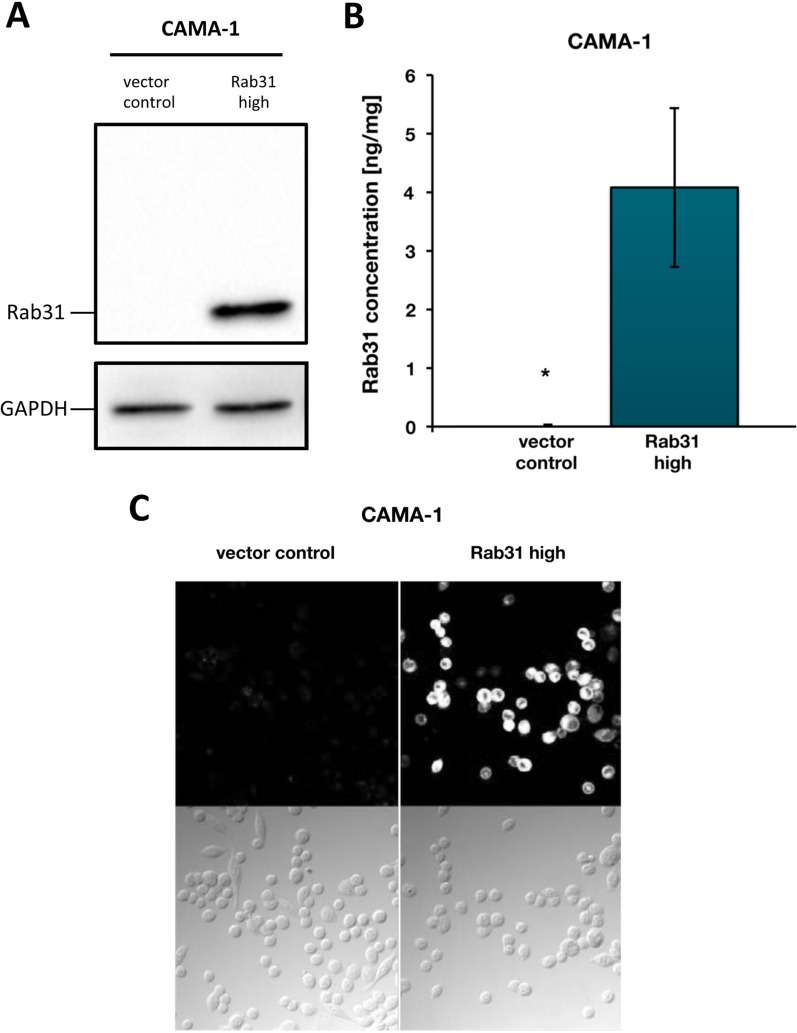
Characterization of CAMA-1 breast cancer cells stably transfected with a Rab31 expression vector. CAMA-1 breast cancer cells were stably transfected with either the plasmid pRcRSV-Rab31 or with the vector alone. **A** Western blot analysis. GAPDH expression served as loading control. **B** Rab31 antigen levels in cell culture lysates, determined by ELISA. At least three experiments were performed in triplicates each. Statistically significant differences (p < 0.05) are indicated by an asterisk. **C** Immunocytochemical analysis of Rab31. Rab31 protein expression is presented in white in the upper panels. The lower panels show transmission images of the cells. Magnification: 20×

The effects of Rab31 overexpression were analyzed in a 3D spheroid model, which more closely mimics the in vivo tumor microenvironment compared to classical 2D cell monolayer cultures. For this, we used CAMA-1 cells, which form spheroids within hydrogel-based 3D cell culture. Rab31 expression in cells which were transfected with a Rab31 expression plasmid or the vector alone was characterized by Western blot analysis (Fig. 1A), ELISA (Fig. 1B), or immunofluorescence (Fig. 1C). Whereas vector control cells do not—or only to a very little extent—express Rab31, upon transfection with the plasmid pRcRSV-Rab31, Rab31 protein expression is distinctly up-regulated. Still, overexpression of Rab31 is within the physiological range because normal platelets display a Rab31 concentration of about 5 ng/mg total protein (Bao et al. 2002). Our ELISA measurements showed a concentration of about 4 ng/mg total protein in CAMA-1 cells (Fig. 1B).

**Fig. 2:**
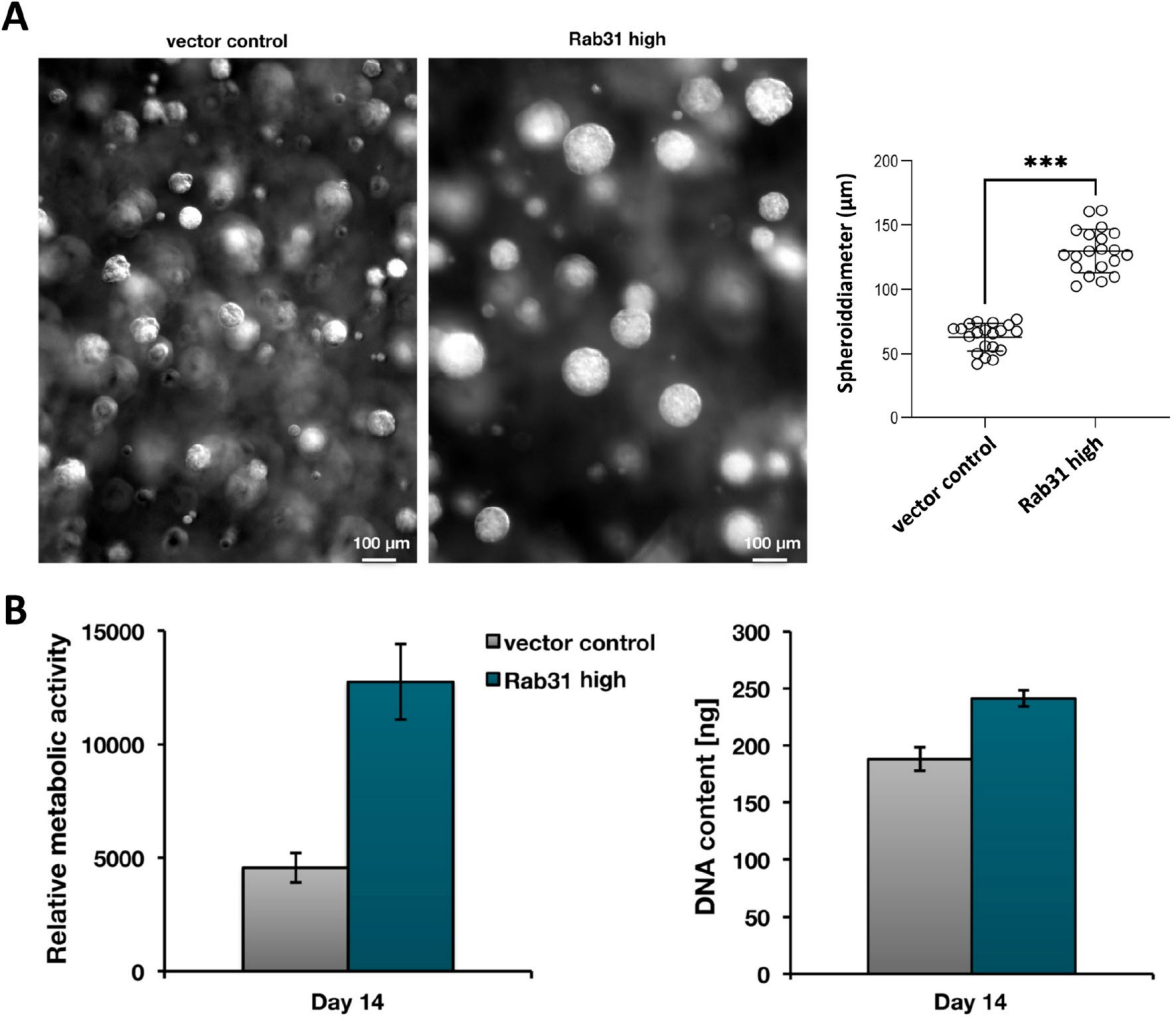
Spheroid formation and proliferation analysis of CAMA-1 Rab31 overexpressing and vector control cells. **A** Both CAMA-1 Rab31 overexpressing and vector control cells formed spheroids in 3D culture, whereby Rab31 overexpressing cells resulted in much larger spheroids indicating a distinctly increased growth rate compared to vector control cells (p < 0.001). **B** Viable cells continuously convert the non-fluorescent substrate AlamarBlue into a fluorescent product (left panel), thereby allowing a quantitative measurement of cell numbers. Since the same cell number was plated out, Rab31 overexpressing cells display a faster growth rate as compared to vector control cells after 14 days. In the CyQuant assay (right panel), the content of DNA is measured by a non-fluorescent substrate, which upon intercalation into genomic DNA exhibits a strong fluorescence. The CyQuant proliferation assay of CAMA-1 cells shows a higher DNA content in Rab31 overexpressing as compared to vector control cells after 14 days, indicating a faster growth rate of Rab31 overexpressing cells. For both the AlamarBlue and the CyQuant assay a representative experiment of three independently performed biological replicates is depicted

When grown in 3D cell culture conditions, Rab31 overexpressing CAMA-1 cells form much larger spheroids than vector-transfected control cells (Fig. 2A), indicating an increased Rab31-dependent cell proliferation rate. In fact, applying both an AlamarBlue assay, that measures the metabolic activity and reflects the number of viable cells, and a CyQuant assay, that is based on determination of the DNA content and indicative of cell proliferation, Rab31 overexpression induced cell proliferation not only in 2D cell monolayers (Grismayer et al. 2012) but also in 3D spheroids (Fig. 2B).

### Identification of differentially expressed genes in Rab31 overexpressing cells

Next, we tested whether Rab31 overexpression in CAMA-1 cells affects expression of other genes involved in EMT-like processes when compared to Rab31 low-expressing vector controls. For initial screening, we used a profiler PCR array (*Human EMT RT*^*2*^* Profiler PCR Array*; Qiagen), which profiles expression of 84 key genes that are involved in EMT or its reciprocal process MET, for Rab31 overexpressing and vector control CAMA-1 cells using two independently generated biological replicates. The cut-off value for significant differential expression was set to > twofold up- or down-regulation. Five genes (*WNT11*, *BMP7*, *TGFB2*, *TIMP1*, and *TGFB1*) were identified as significantly downregulated in Rab31 overexpressing cells in both experiments, whereas four genes (*TFPI2*, *VCAN*, *STEAP1*, and *PLEK2*) were consistently upregulated (Table 1). The strongest effects were seen for *TGFB1*, encoding TGF-ß1, displaying a > 25-fold down-regulation in Rab31 overexpressing cells compared to vector controls.

**Table 1:** Differentially expressed EMT-related candidate genes in Rab31 overexpressing and vector control cells identified by profiler PCR array analysis

Gene	Protein
Downregulated in Rab31 overexpressing CAMA-1 cells	
*TGFB1*	Transforming growth factor, beta 1
*TGFB2*	Transforming growth factor, beta 2
*TIMP-1*	Tissue inhibitor of metalloproteases 1
*BMP7*	Bone morphogenetic protein 7
*WNT11*	Wnt family member 11
Upregulated in Rab31 overexpressing CAMA-1 cells	
*VCAN*	Versican
*TFPI2*	Tissue factor pathway inhibitor 2
*STEAP1*	STEAP family member 1
*PLEK2*	Pleckstrin 2

The mRNA of the depicted genes was at least twofold differentially regulated in Rab31 overexpressing compared to vector control cells in two independent biological replicates applying the *Human EMT RT*^*2*^* Profiler PCR Array* (Qiagen)

### Validation of differential TGF-β1 mRNA expression in Rab31 overexpressing cells

**Fig. 3:**
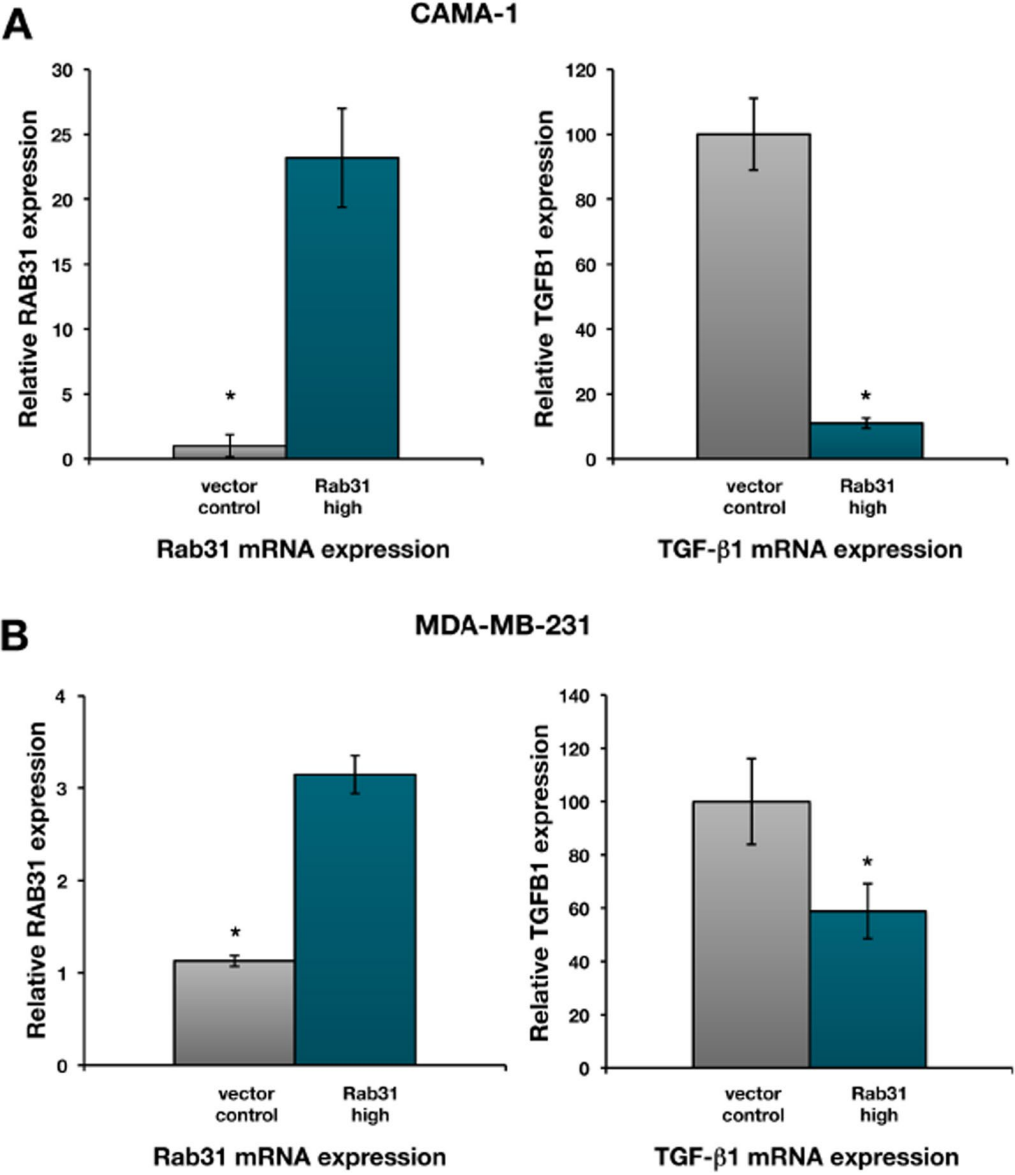
Rab31 and TGF-ß1 mRNA expression in Rab31 overexpressing and vector control cells. Relative Rab31 and TGF-ß1 mRNA expression levels, normalized to the HPRT1 mRNA expression levels, in CAMA-1 (**A**) and MDA-MB-231 (**B**) breast cancer cells, respectively. Three experiments were performed in triplicates each. Statistically significant differences (p < 0.05) are indicated by an asterisk

**Fig. 4:**
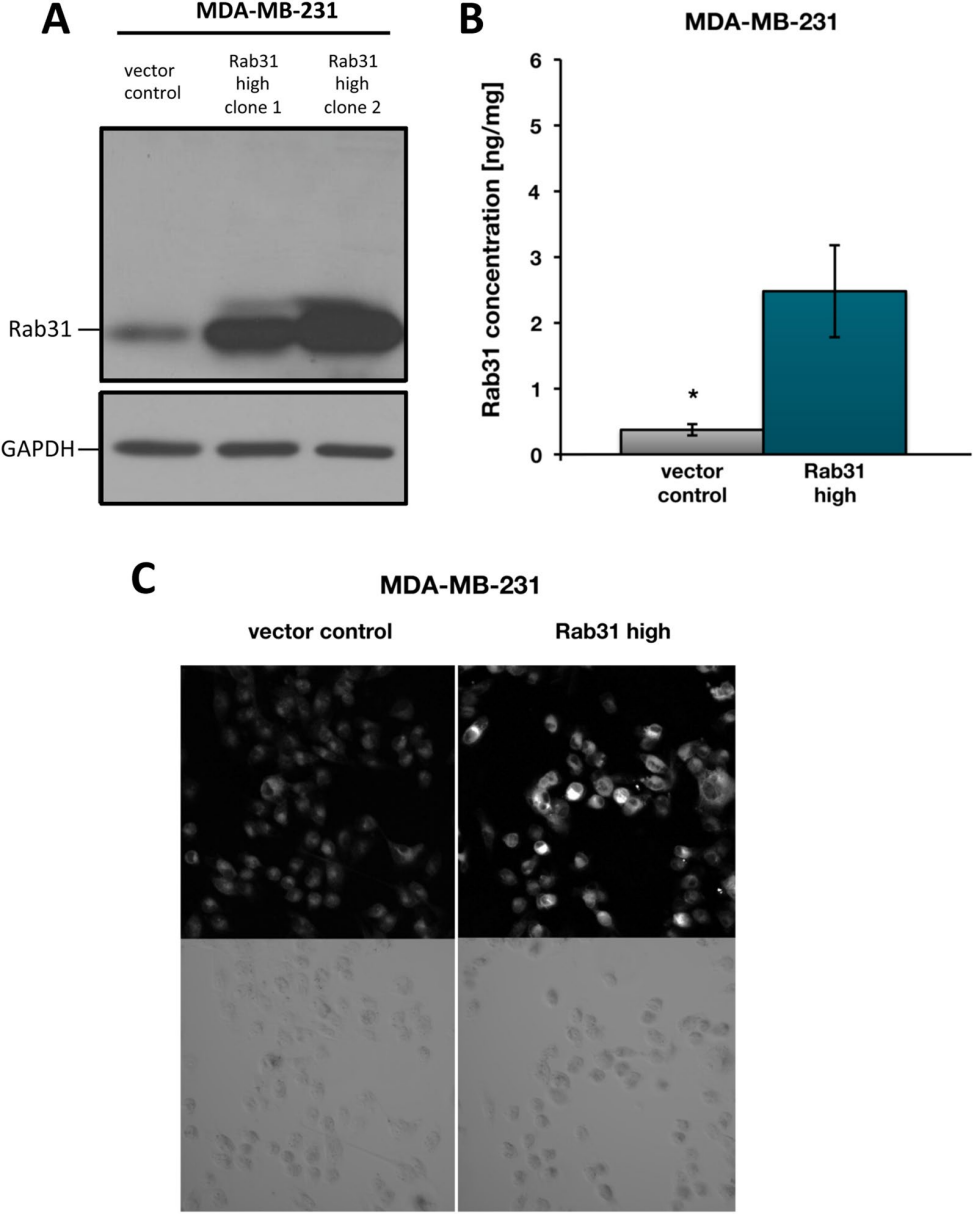
Characterization of MDA-MB-231 breast cancer cells stably transfected with Rab31. MDA-MB-231 breast cancer cells were stably transfected with either the plasmid pRcRSV-Rab31 or with the vector alone. **A** Western blot analysis. In addition to vector control cells, the Western blot shows two independently selected Rab31-overexpressing MDA-MB-231 cell clones. Clone 2 was used for all further experiments described in the manuscript. GAPDH expression served as loading control. **B** Rab31 antigen levels in cell culture lysates, determined by ELISA. At least three experiments were performed in triplicates each. Statistically significant differences (p < 0.05) are indicated by an asterisk. **C** Immunocytochemical analysis of Rab31. Rab31 protein expression is presented in white in the upper panels. The lower panels show transmission images of the cells. Magnification: 20×

In order to validate downregulation of *TGFB1* gene expression in response to Rab31 overexpression in CAMA-1 cells, TGF-ß1 (and Rab31) mRNA levels were quantified by an independent qPCR assay (TaqMan gene expression assay; Life Technologies). Again, a strong more than 20-fold downregulation of *TGFB1* gene expression was observed in response to increased Rab31 expression (Fig. 3A). In addition to CAMA-1 cells, we also analyzed a second breast cancer cell line, MDA-MB-231 (Fig. 4), which expresses higher basal Rab31 protein levels compared to CAMA-1 cells. In MDA-MB-231 cells, stable transfection of the Rab31 expression plasmid pRcRSV-Rab31 resulted in expression levels of about 2.5 ng/mg total protein (Fig. 4B), which reflects a fivefold increased protein level compared to vector control MDA-MB-231 cells. Quantitation of Rab31 and TGF-ß1 mRNA levels again showed that overexpression of Rab31 leads to a significant downregulation of TGF-ß1 mRNA expression in MDA-MB-231 (Fig. 3B), albeit to a lower extent than observed in CAMA-1 cells.

### Analysis of TGF-β1 protein expression and activity in Rab31 high- versus low-expressing cells

**Fig. 5:**
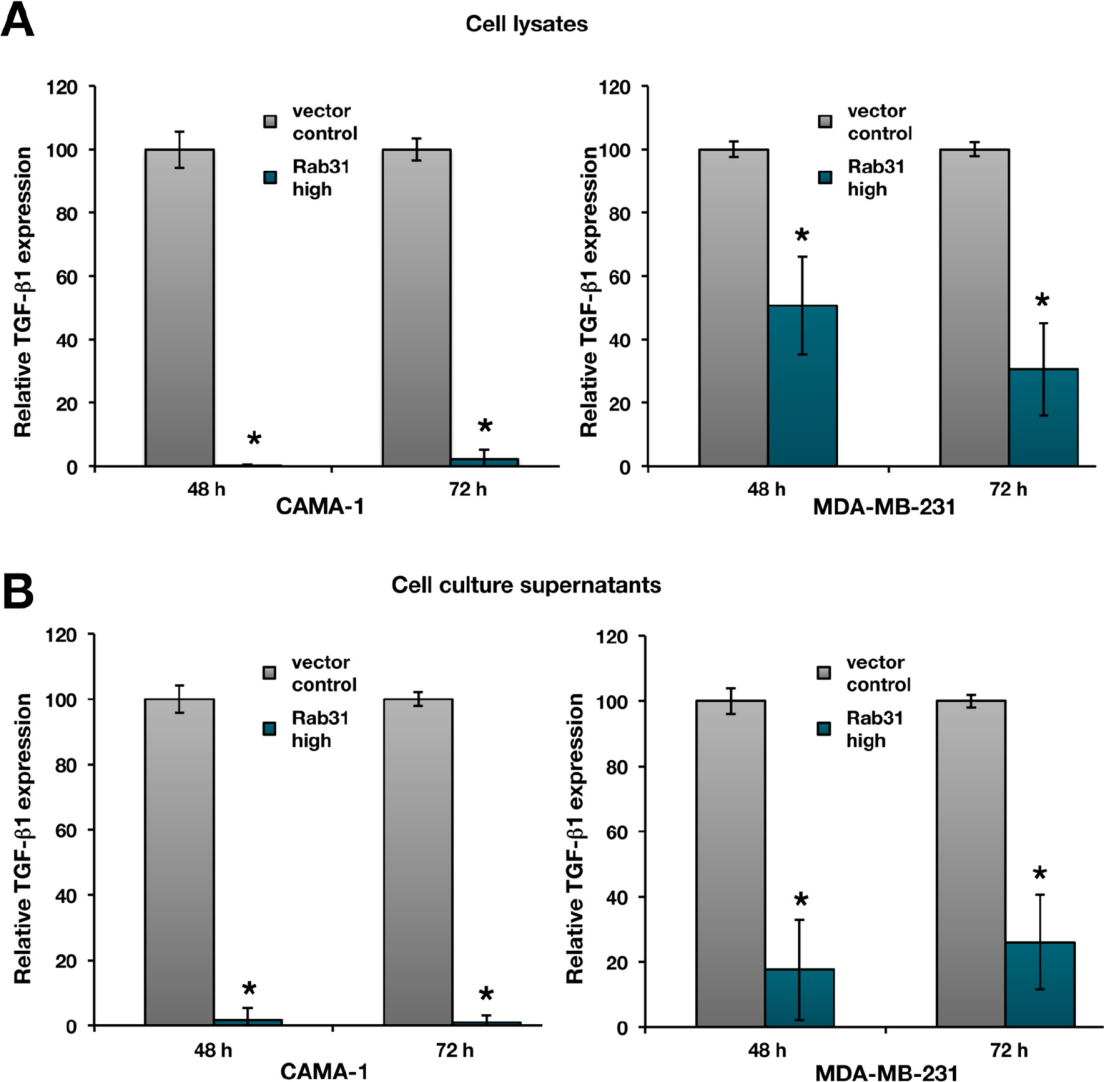
Relative TGF-ß1 antigen levels in cell culture supernatants and cell lysates from Rab31 low and high expressing cells. Cells were grown for 48 h or 72 h in FCS-free medium. TGF-β1 antigen levels from cell lysates (**A**) or from cell culture supernatants (**B**) were determined by ELISA and normalized to the total protein content of the respective sample. Three experiments were performed in triplicates each. Statistically significant differences (p < 0.05) are indicated by an asterisk

**Fig. 6:**
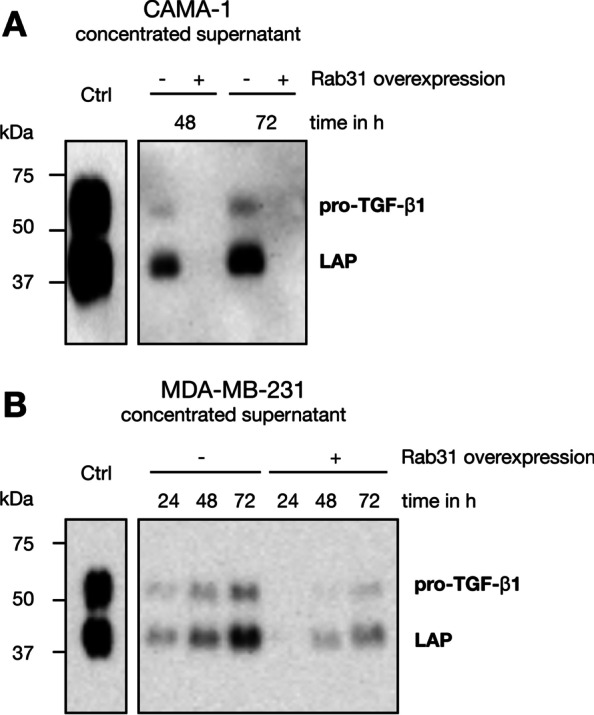
Western blot analysis of TGF-ß1 in cell culture supernatants of Rab31 low- and high-expressing cells. Concentrated cell culture supernatants (concentration factor 1:16; the total protein content of the supernatants was used for normalization) of CAMA-1 (**A**) or MDA-MB231 (**B**) Rab31 overexpressing and vector control cells, respectively, were reacted with a TGF-ß1 specific antibody. The signals obtained match the expected size for pro-TGF-ß1 and its cleavage product LAP, respectively

In order to evaluate TGF-ß1 regulation by Rab31 overexpression at the protein level, total TGF-ß1 antigen levels were assessed in whole cell lysates using an ELISA-based approach. We found considerably reduced TGF-ß1 protein levels in both CAMA-1 and MDA-MB-231 Rab31-overexpressing cells, which was more pronounced in the CAMA-1 cells (Fig. 5A). In parallel, total TGF-ß1 levels were determined in cell culture supernatants by ELISA (Fig. 5B) and by Western blot, using an antibody directed to both pro-TGF-ß1 and LAP, which corresponds to the pro-domain of TGF-ß1 (Fig. 6). Again, we found distinctly lower secreted TGF-ß1 levels in Rab31 overexpressing cells.

**Fig. 7:**
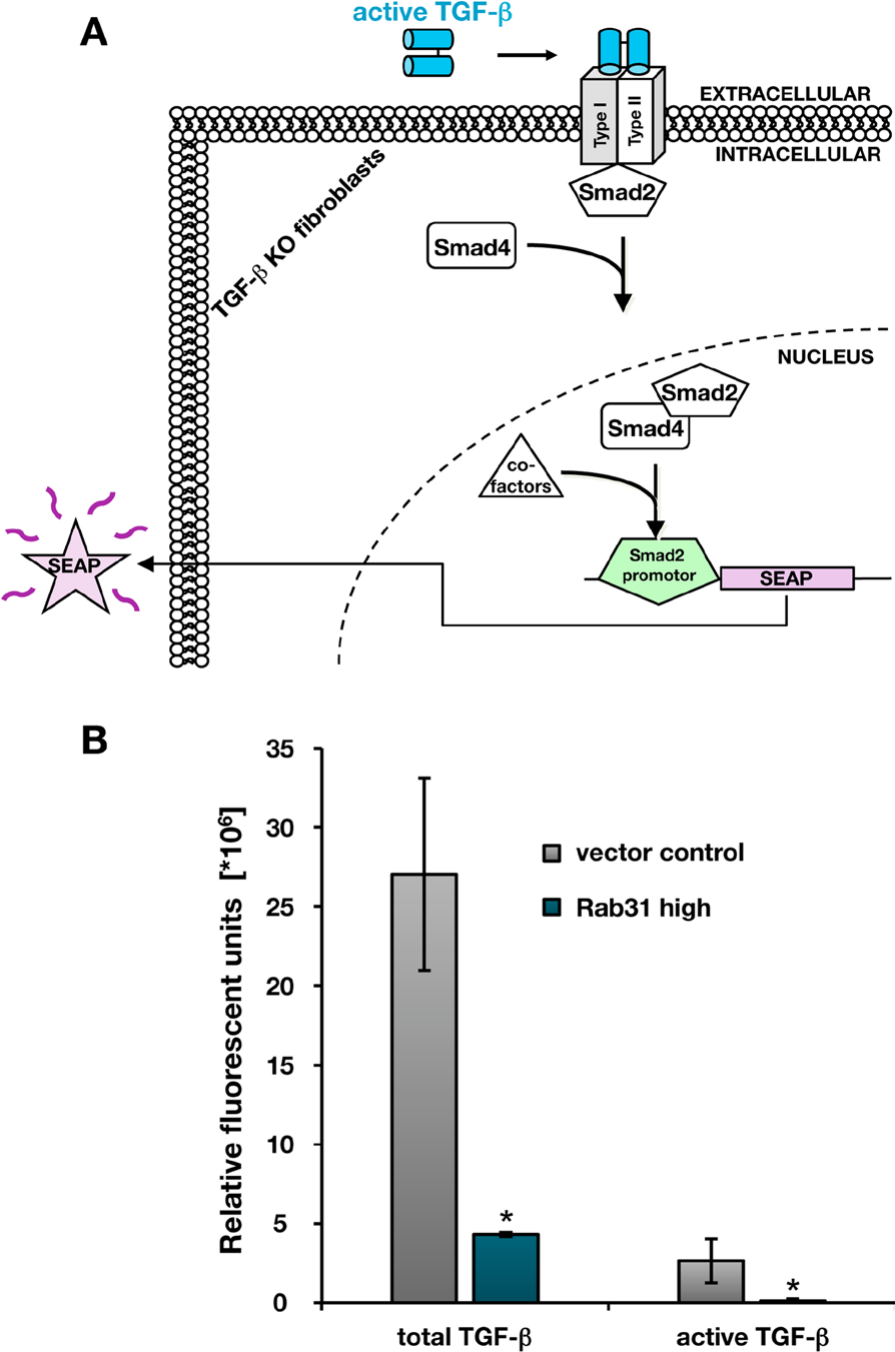
TGF-ß activity assays of cell culture supernatants from Rab31 overexpressing and vector control cells. **A** Outline of the human TGF-ß activity assay. Concentrated supernatants (1:16) from CAMA-1 Rab31 overexpressing and vector control cells, were added to murine TGFB1−/− fibroblasts transfected with a TGF-ß reporter plasmid. This plasmid encodes secreted alkaline phosphatase (SEAP) under the control of SMAD-binding elements (SBE). SEAP activity is then determined by a chemiluminescent reaction. **B** CAMA-1 cell culture supernatants were heat-activated to measure total TGF-ß levels (left panel), or left untreated to measure active TGF-ß levels (right panel). Statistically significant differences (p < 0.05) are indicated by an asterisk

Since neither ELISA nor Western blot analyses using antibodies directed to mature TGF-ß were sensitive enough to detect active TGF-ß1, we used a highly sensitive, cell-based bioassay (Fig. 7A) that allows detection of human TGF-ß levels as low as 1 pg/ml versus 31.2 pg/ml by the ELISA approach (Beaufort et al. 2014; Broekelmann et al. 2020; Johnston et al. 2017; Tesseur et al. 2006). Concentrated supernatants from CAMA-1 Rab31 and vector transfectants were left untreated to measure active TGF-ß levels or heated to 65 °C to activate the pool of latent TGF-β in order to allow the determination of the total TGF-ß content. In line with the results obtained by ELISA and Western blot analyses, lower total TGF-ß levels were detected in the supernatants from Rab31 overexpressing cells when compared to vector controls. Similarly, active TGF-ß levels were strongly reduced upon Rab31 overexpression (Fig. 7B).

### Identification of further differentially expressed genes using a TGF-ß/BMP signaling profiler array

To further illuminate the relationship between Rab31 expression and the TGF-ß signaling axis, another profiler PCR array was selected (*Human TGF-ß/BMP RT*^*2*^* Profiler PCR Array*; Qiagen) focusing on a panel of genes related to TGF-ß signaling including TGF-ß superfamily ligands and receptors, transcription factors (e.g. SMADs) and TGF-ß targets. Following the strategy applied for the initial screen using the EMT-related profiler PCR array, CAMA-1 cells were examined twice using independently generated biological replicates. Here, 12 out of 84 mRNAs were at least twofold reduced and 7 mRNAs were > twofold increased upon Rab31 overexpression in both experiments (Table 2). Three genes, *TGFB1*, *TGFB2*, and *BMP7*, are included in both the EMT (Table 1) and TGF-ß1/BMP (Table 2) profiler PCR array and were identified as differentially regulated candidates in both arrays. Whereas *TGFB1* and *TGFB2* were down-regulated upon Rab31 overexpression, expression of *TGFB3* (present on the TGF-ß1/BMP array only) was not affected.

**Table 2:**
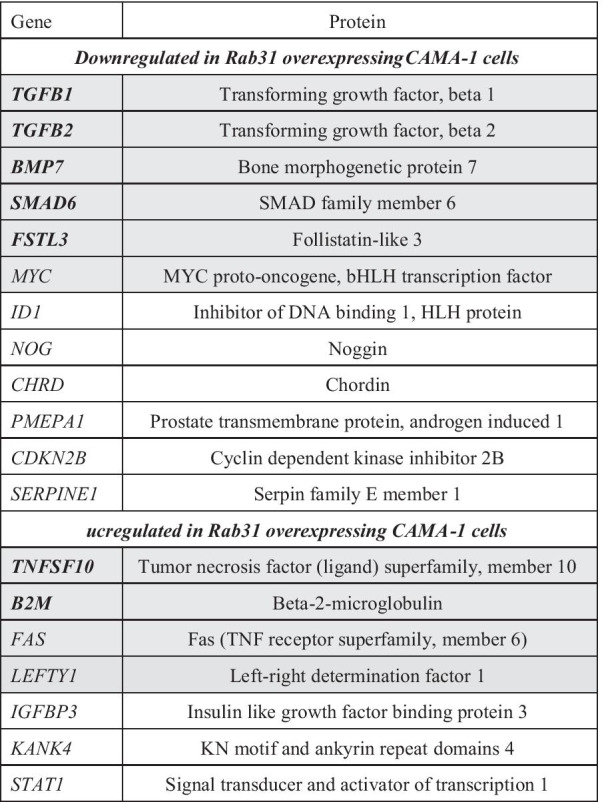
Differentially expressed TGF-ß pathway-related candidate genes in Rab31 overexpressing and vector control cells identified by profiler PCR array analysis

The mRNA of the depicted genes was at least twofold differentially regulated in Rab31 overexpressing compared to vector control cells in two independent biological replicates applying the *Human TGF-β/BMP RT*^*2*^* Profiler PCR Array* (Qiagen). The genes with grey background were further analyzed by using Roche Universal Probe Library-based qPCR assays, whereby the genes in bold were validated as differentially expressed genes

### Validation of differential mRNA expression of selected candidates in Rab31 overexpressing cells

**Fig. 8:**
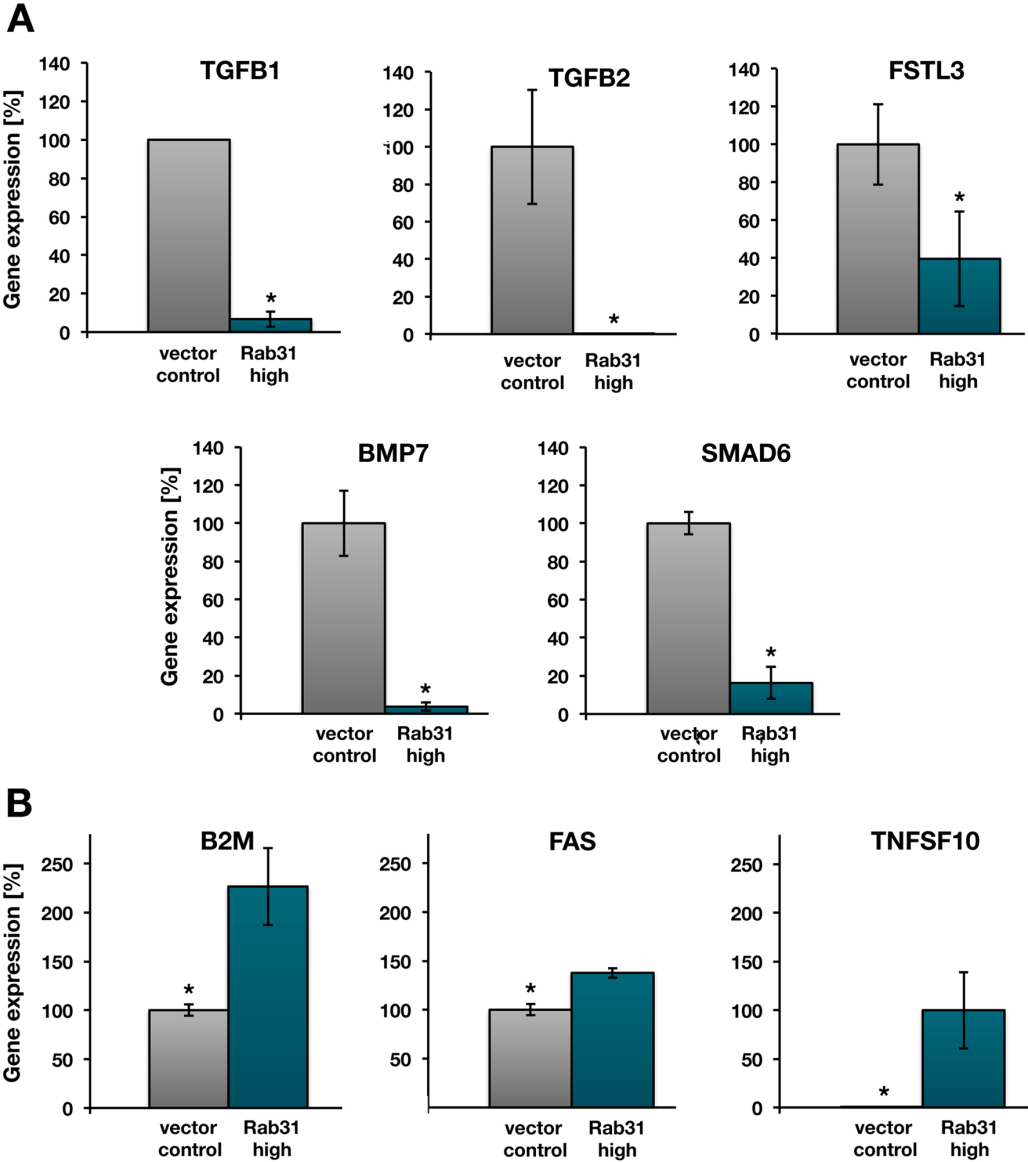
Validation of differential gene expression in Rab31 overexpressing and vector control cells. **A** Validation of the significant, more than twofold, down-regulation of the five genes *TGFB1*, *TGFB2*, *FSTL3*, *BMP7*, and *SMAD6* in Rab31 overexpressing and vector control cells. **B** Quantitative determination of the *B2M*, *FAS*, and *TNFSF10* mRNA levels CAMA-1 cells. The significant, more than twofold up-regulation of the genes *B2M* and *TNFSF10* in Rab31 overexpressing and vector control cells is in line with the profiler PCR array analyses (see Table 2). In case of *FAS*, although significant, only an approx. 1.4-fold increase is observed in Rab31 overexpressing over vector control cells. In all cases, except for *TNFSF10*, the mRNA levels from vector control cells were set to 100% and the relative mRNA levels in the corresponding Rab31 overexpressing cells was calculated accordingly. For TNFSF10 expression, the mRNA levels of Rab31 overexpressing cells were set to 100%. All samples were normalized to HPRT1 expression level. Three experiments were performed in triplicates each. Statistically significant differences (p < 0.05) are indicated by an asterisk

To validate differential expression of selected candidates in Rab31 overexpressing and vector control CAMA-1 cells, we applied independent qPCR assays (using Roche Universal Probe Library probes). These assays use different probes and primers than those used in the profiler PCR arrays. In addition to *TGFB1*, the following 9 genes were selected: *TGFB2*, *BMP7*, *SMAD6, FSTL3* and *MYC*, which represent candidates for downregulation, and *TNFS10, B2M*, *FAS* and *LEFTY*, representing potentially upregulated genes upon Rab31 overexpression (Table 2). Of these candidate genes, 7 (*TGFB1, TGFB2*, *BMP7*, *SMAD6*, *FSTL3*, *TNFSF10* and *B2M*) were at least twofold differentially expressed (Fig. 8; see also genes indicated in bold in Table 2). In case of *MYC* and *LEFTY*, even after 40 cycles no PCR product was detected using the TaqMan system, which either may indicate that these candidate genes represent false-positive genes within the profiler PCR array screen or the TaqMan assay is not sensitive enough to show differential expression. *FAS* was only slightly upregulated (about 1.4-fold) and does not meet the criterion of at least twofold difference in the mRNA levels between Rab31 overexpressing and vector control cells to represent a differentially expressed gene. Overall, these data clearly demonstrate that TGF-ß expression levels and related proteins involved in the TGF-ß signaling pathway are—at least in part—under the control of Rab31 in a dose-dependent manner.

## Discussion

The members of the Rab protein family are distributed within distinct cellular compartments and represent major regulators of vesicle trafficking in many fundamental processes (Homma et al. 2020). In the activated state, Rab proteins promote downstream signaling by interacting with various effector proteins (Zhen and Stenmark 2015). Rab5 cycling between the GTP and GDP forms may influence the length and intensity of TGF-ß/activin signaling cascades (Panopoulou et al. 2002). Dysregulated expression of Rab-coding genes leads to distinct variations in biological functions and diseases (Krishnan et al. 2020). In cancer, Rab proteins contribute either to the suppression or promotion of tumor development, growth and metastasis. To this end, elevated Rab31 expression in breast cancer tissue is associated with poor patient prognosis (Kotzsch et al. 2008, 2017) and affects several tumor-relevant processes: increased Rab31 expression in breast cancer cells leads to enhanced proliferation concomitant with reduced cell adhesion and decreased invasion. (Grismayer et al. 2012). Knockdown of Rab31 expression in glioblastoma cells suppressed tumor growth in a nude mouse model (Pan et al. 2016). Although various pathways have been proposed for Rab31-induced cell proliferation (Tang et al. 2018; Yu et al. 2019), the exact mechanism of how Rab31 modulates tumor growth remains elusive. In the present study, we extended the analyses of the Rab31 effects on breast cancer cell proliferation using a 3D cell culture model. 3D cell culture systems are miniaturized tumor tissue/microtumor-like models that allow the growth of spheroids and recapitulate the physico-chemical properties of the cell–cell and cell–matrix interactions that occur in patient tissues (Hutmacher et al. 2010). These aspects contrast conventional 2D systems, which are limited by a uniform monolayer of cells cultured on a plastic surface. The simplistic 2D cell culture does not provide the multiple stimuli nor recapitulate the complexity of interactions that characterize the tumor microenvironment, and therefore, are determinant for the selection of a suitable 3D approach. We found that Rab31 overexpressing cells did not only exhibit increased proliferation rates but led also to the formation of larger spheroids as compared to control cells over 14 days. This new result is in line with our previously reported proliferation data using a conventional 2D system, in which Rab31 also enhanced cell proliferation over 3–4 days. The advantages of our herein reported hydrogel-based 3D assay are that it is not only better representing the tumor microenvironment but also allows for long-term measurements of cell behavior (e.g., spheroid formation, proliferation, metabolic activity).

Tumor growth and metastasis are characterized by uncontrolled cell proliferation, adhesion and invasion (Ribatti et al. 2020). These cell functions are strongly associated with both EMT and its reciprocal process MET. MET occurs in various stages of embryogenesis and is also used by cancer cells to establish micro-metastases and secondary lesions (Thiery 2009). However, the understanding of the events required to regulate EMT/MET-like processes in metastasis is still limited. A number of Rab-GTPases have been shown to regulate cancer-associated EMT-like programs. As such, overexpression of Rab25 contributed to bladder cancer metastasis through the induction of EMT (Zhang et al. 2013). Studies using Rab3D knockdown or overexpression in breast cancer cells strongly suggest that this Rab-GTPase regulates EMT via the activation of the Akt/GSK-3β/Snail pathway (Yang et al. 2015). Moreover, small GTPases play essential roles in TGF-ß-induced actin remodeling and cytoskeleton rearrangement, which facilitate processes like EMT (Kardassis et al. 2009; Ungefroren et al. 2018).

Previously, we reported that EMT/MET-associated cell functions (cell proliferation, adhesion, and invasion) are influenced by the Rab31 expression level (Grismayer et al. 2012). Here, we used a profiler PCR array system to assess the regulation of genes involved in EMT/MET in Rab31 overexpressing CAMA-1 breast cancer cells versus the respective vector transfectants displaying only low endogenous Rab31 levels. We identified 9 differentially expressed genes, with the gene encoding TGF-ß1 displaying the most pronounced difference (> 25-fold downregulation in Rab31-overexpressing versus control cells). Because most of the other candidate genes have been associated with the TGF-ß signaling pathway, we focused on TGF-ß1 for further analysis. On the protein level, a strong reduction of TGF-ß1 was observed in CAMA-1 Rab31 overexpressing cells compared to the vector control in both cell lysates and cell culture supernatants. Similar results were obtained with a second breast cancer cell line, MDA-MB-231. These cells, however, showed a moderate reduction of TGF-ß1 upon Rab31 overexpression, probably due to the intrinsic Rab31 levels already present in the parental cells. In line with these observations, comparing cell lines with different endogenous Rab31 levels, the strongest effects of Rab31 overexpression on elevated proliferation were detected in breast cancer cell lines not showing any detectable Rab31 expression like CAMA-1 (Grismayer 2012). In addition, there may be differential regulatory effects of ER expression, since MUC1-C in complex with ERα activates Rab31 expression and, in turn, Rab31 increases MUC1-C levels in a positive feedback auto-inductive loop (Jin et al. 2012). This may potentiate the effect of Rab31 overexpression on TGF-ß1 reduction in ER-positive CAMA-1 cells. Most importantly, our present results demonstrate that not only protein production but also activity of TGF-ß1 was considerably reduced as shown by our highly sensitive cell-based bioassay. To complement our findings, future work should evaluate the impact of Rab31 overexpression in tumor cells on TGFβ1-related signaling pathways, including both SMAD-dependent and non-canonical signaling cascades e.g., Erk, JNK/p38 MAPK, small GTPase and PI3K/Akt.

TGF-ß signaling is an important pathway in cancer progression, which mediates both tumor-suppressive and pro-oncogenic effects (Meulmeester and Ten Dijke 2011; Syed 2016; Wakefield and Roberts 2002; Zu et al. 2012). The earliest event described in TGF-ß-induced EMT is the activation of a regulatory enzyme of the Ras family, namely RhoA (Tavares et al. 2006; Yang and Weinberg 2008). Moreover, TGF-ß induces EMT in co-operation with Ras GTPase signaling, which leads to a more invasive phenotype in response to stimulation by growth factors (Oft et al. 2002; Ungefroren et al. 2018). During EMT, cells elongate and secrete proteases, which degrade the extracellular matrix facilitating their invasion. Cancer cells can form actin-rich adhesion structures, so-called invadopodia, and, by this, are able to alter the tumor microenvironment. TGF-ß stimulates invadopodia formation in numerous cancer cells (Augoff et al. 2020). In vivo, tumor cells use invadopodia-like structures for intravasation by polarized secretion of proteases (Eddy et al. 2017). Strikingly, in these processes, Rab-GTPases, e.g. Rab7, Rab5a and Rab4, are involved via regulation of the vesicular transport (Alli-Balogun et al. 2016; Frittoli et al. 2014; Monteiro et al. 2013). Noll and co-workers (2019) even showed that Rab4 downregulation inhibited the matrix degradation of TGF-ß-treated breast cancer cells. Rab proteins take also part in internalization and recycling of the TGF-ß receptor via vesicle transport, which provides a means to regulate the number of surface receptors (Porther and Barbieri 2015).

We previously showed that Rab31 overexpression attenuates invasion and enhances proliferation of breast cancer cells through inhibition of the EMT-like phenotype and the switch to the more proliferative MET-like phenotype (Grismayer et al. 2012). This suggests that the observed drop of TGF-ß in these cells may be critical in this process. In order to further elucidate the Rab31 effects on the TGF-ß signaling pathway, we extended our search for differentially regulated genes in Rab31 overexpressing versus control cells and performed a profiler PCR array with a focus on genes of the TGF-ß superfamily. In addition to *TGFB1*, several other candidate genes including *TGFB2*, *BMP7* and *SMAD-6*, were identified and subsequently validated to be selectively up- or downregulated upon cellular Rab31 overexpression. Our findings indicate that Rab31, depending on its expression level, has a crucial impact on the expression of various components of the TGF-ß signaling pathway in breast cancer cells.

Most of our identified differentially expressed genes, including *TGFB1*, display "chameleon-like" activity, i.e.*,* the encoded proteins can exert opposite effects in different settings, e.g.*,* different cell lines or tumor samples. This so-called antagonistic duality of cancer genes certainly depends on the genetic context and the tumor microenvironment, which lead to (in)activation of signaling pathways within a complex network and to the seemingly paradox, opposite responses to the same protein (Stepanenko et al. 2013). In our future studies, we will investigate the relation between the expression patterns of the various identified factors and phenotypic changes mediated by the modulation of Rab31 levels.

TGFβ1 and EMT are key regulators of tumor cell migration, invasion, and metastasis (Pang et al. 2016; Chen et al. 2017). However, TGFβ1 also exerts well-described tumor suppressive functions (cytostasis, differentiation, apoptosis, suppression of inflammation and stroma-derived mitogens) in early stages (Massague 2008, Cantelli et al. 2017). Overexpression of Rab31 in breast cancer cells, resulting in downregulation of TGF-ß, may lead to a deregulation of the balance between cell cycle arrest and cell proliferation in favor of tumor growth. A study by Agajanian and co-workers (2015) showed that the Src-regulator PEAK1 mediates the shift of TGF-ß responses from an anti-proliferative to pro-tumorigenic function, highlighting the importance of signaling pathway crosstalk during cancer progression. Hence, cancer cells must initially find a way to escape growth inhibition and cell death regulated by the TGF-ß pathway. One possible mechanism is the truncation of the pathway via receptor-inactivating mutations and, consequently, silencing of TGF-ß signaling. Another mechanism may be to selectively tune down the tumor-suppressive arm of the pathway either by mutations within the cascade or by inhibition caused by other signaling cascades (Bierie and Moses 2009; Ikushima and Miyazono 2010; Massagué 2008; Morrison et al. 2013). Repression of TGF-ß by Rab31 overexpression may be part of the later mechanism to dampen the tumor-suppressing activities of the TGF-ß pathway. During invasion and metastasis, Rab31-triggered downregulation of TGF-ß may be lost, since, e.g., elevated levels of TGF-β—leading to an EMT-like phenotype of the cancer cells—have been found at invasive fronts in human breast cancer tissues (reviewed in Morrison et al. 2013). All in all, it seems reasonable that elevated Rab31 levels mediate anti-EMT-like activities which promote initial primary tumor growth as well as re-establishment of metastases at a distant site. This may well explain also the clinical finding that elevated Rab31 levels, determined in primary tumor tissue, are associated with poor patient prognosis.

## Conclusions

In the present study, we demonstrate that Rab31 is a potent modulator of the expression of TGF-ß and other components of the TGF-ß signaling pathway in breast cancer cells. Its impact on TGF-ß expression in the primary tumor or in the distant metastases resulting in tumor growth may explain why high Rab31 levels in tumor tissue are related to poor patient prognosis.

## Data Availability

All data generated or analyzed during this study are included in this article.

## Abbreviations

BSA: Bovine serum albumin
BMP: Bone morphogenetic proteins
CLSM: Confocal laser scanning microscopy
DMEM: Dulbecco’s modified Eagle medium
ELISA: Enzyme-linked immunosorbent assay
EMT: Epithelial-to-mesenchymal transition
ER: Estrogen receptor
FCS: Fetal calf serum
GDP: Guanosine diphosphate
GTP: Guanosine triphosphate
HPRT: Hypoxanthine guanine phosphoribosyl transferase 1
LAP: Latent-associated protein
MET: Mesenchymal-to-epithelial transition
MUC1-C: Mucin-1
PBS: Phospate-buffered saline
PCR: Polymerase chain reaction
qPCR: Quantitative PCR
Rab: Ras-related protein in brain
RT: Room temperature
SEAP: Secreted alkaline phosphatase
TBS: Tris-buffered saline
TBS-T: TBS containing 0.1% (v/v) Tween-20
TGF-ß: Transforming growth factor-ß
TMB: 3,3’,5,5’-Tetramethylbenzidine
